# Sex expression and inbreeding depression in progeny derived from an extraordinary hermaphrodite of *Salix subfragilis*

**DOI:** 10.1186/1999-3110-55-3

**Published:** 2014-01-14

**Authors:** Teruyoshi Nagamitsu, Norihiro Futamura

**Affiliations:** Forestry and Forest Products Research Institute (FFPRI), Tsukuba, 305-8687 Japan

**Keywords:** Dioecy, Hermaphroditism, Inbreeding depression, *Salix subfragilis*, Selfing, Sex expression

## Abstract

**Background:**

An extraordinary hermaphrodite of dioecious willows provides us an opportunity to examine the inheritance of sex expression and the magnitude of inbreeding depression using a progeny assay of the hermaphrodite.

**Results:**

We indentified 165 progeny of an open-pollinated hermaphrodite of *Salix subfragilis* as siblings selfed (Self) or crossed with another hermaphrodite (Cross_H) or a male (Cross_M) using microsatellite genotypes. There were more selfed progeny (110 in Self) than outcrossed progeny (31 in Cross_H and 24 in Cross_M), suggesting the absence of barriers to selfing in the maternal hermaphrodite. The sex ratio (female:male:hermaphrodite) of the progeny differed among the sibling groups (27:17:66 in Self, 3:16:12 in Cross_H and 9:8:7 in Cross_M). Nearly half of the selfed progeny were hermaphrodites, suggesting that an identical combination of parental alleles in progeny reproduced the hermaphroditism of the parent. We measured fitness components of growth (stem height and basal area), survival and fertility (pollen germination proportion, number of ovules and seed set). The magnitudes of inbreeding depression in growth and survival (0.29-0.70) were higher than those in fertility (0.00-0.16).

**Conclusions:**

The findings suggest a genetic basis of extraordinary hermaphroditism and substantial inbreeding depression in survival and growth in the dieocious *S. subfragilis*.

**Electronic supplementary material:**

The online version of this article (doi:10.1186/1999-3110-55-3) contains supplementary material, which is available to authorized users.

## Background

Willows (genus *Salix*, Salicaceae) are promising materials for short-rotation forestry for renewable energy. Breeding programs for willows require a basic knowledge of the reproduction and genetics of the genus *Salix* (Karp et al., 2011). Dioecy is a noteworthy character of willows, which consist of females with pistllate catkins and males with staminate catkins. However, extraordinary polygamy has been found in some individuals, which bear catkins of both female and male flowers in various compositions (Smith, 1940; Mosseler and Zsuffa, 1989; Falinski, 1998; Rottenberg, 2007). Furthermore, hermaphroditic flowers with pistils and stamens were discovered in *S. martiana* (Rohwer and Kubitzki, 1984). Such bisexual individuals provide us an opportunity to investigate the mechanism of sex determination and the inheritance of sex expression. If self-fertilization occurs, the presence of self-incompatibility and the magnitude of inbreeding depression can be evaluated in normally dioecious willows. However, sex expression and inbreeding depression using progeny derived from such bisexual willows have been rarely studied.

The mechanism of sex determination is unknown in willows. It cannot be explained by the presence of sex chromosomes without assuming multiple independent loci for sex determination in *S. viminalis* (Alström-Rapaport et al., 1997). In poplars (genus *Populus*, Salicaceae), which have a nuclear genome with a structure similar to that of *Salix* (Hanley et al., 2006), genetic factors of sex determination have been investigated using genetic linkage maps in four pedigrees (Gaudet et al., 2007; Yin et al., 2008; Pakull et al., 2009;2010). These studies have shown that the sex determination locus appears to be sited in the same linkage group. However, the positions of the sex determination locus in the linkage group and the heterogametic sex differ among the pedigrees. Thus, the mechanisms of sex determination are divergent even in poplars (Paolucci et al., 2010) although the linkage group might be an incipient sex choromosome in the poplar genomes (Yin et al., 2008). In the evolution of sex chromosomes, a ressesive allele for male sterility and a dominant allele for female sterility at closely linked loci are thought to play an important role in the transition from hermaphroditism to dioecy through gynodioecy (Charlesworth and Charlesworth, 1978). In this process, a mutation at the dominant allele for female sterility or a recombination of the two sterility loci results in reversion to hermaphroditism (Charlesworth, 2002). If the extraordinary expression of hermaphroditism in willows has a genetic basis, the progeny will inherit the hermaphroditic expression, and the sex ratio will depend on the segregation of parental alleles. The inheritance of hermaphrodite expression may suggest the mechanism of sex determination in willows.

Willows are dioecious and thus are exclusively outcrossing. Progeny derived from selfing in hermaphroditic willows will exhibit a substantial magnitude of inbreeding depression because predominantly outcrossing species of woody plants tend to accumulate deleterious mutations (Husband and Schemske, 1996; Duminil et al., 2009). In willows, however, the number of lethal equivalents (2.0 to 3.6) found in *S. viminalis* estimated from full-sib crosses was lower than that in most conifers (>8) (Kang et al., 1992). The magnitude of inbreeding depression tends to differ among various fitness traits depending on the occurance of deleterious mutations and selection against the mutations (Charlesworth and Willis, 2009). A meta-analysis of inbreeding depression in various fitness components, such as embryogenesis, growth, survival and fertility, showed larger differences in inbreeding depression among the components in self-compatible species than among those in self-incompatible species (Angeloni et al., 2011). This fact is interpreted as different opportunities for selection against deleterious mutations among fitness components because deleterious mutations expressed at the early life-cycle stages are more frequently purged from inbred populations than those at late life-cycle stages (Husband and Schemske, 1996). A consistent trend of inbreeding depression during the life-cycle stages, however, was not observed (Angeloni et al., 2011). These findings suggest that the magnitude of inbreeding depression is difficult to predict because it varies among fitness traits, probably due to differential mutation and selection specific to individual traits.

In this study, we investigated the inheritance of sex expression and the magnitude of inbreeding depression using selfed and outcrossed progeny of an extraordinary hermaphrodite discovered in *S. subfragilis*. First, we examined differences in sex expression based on the sex ratio among the sibling progeny. Second, we measured fitness components in survival, growth and fertility and compared the magnitudes of inbreeding depression among these components.

## Methods

### Plant materials

*Salix subfragilis* Andersson (Salicaceae) is a dioecious tree or shrub that reaches a height of 3-10 m. This species is native to Japan, Korea, the Russian Far East and northern China. An extraordinary hermaphrodite of this species was found in 1999 in Hokkaido (43°8'N, 142°24'E), nothern Japan (Kurahashi and Kimura, 2002). Four offspring derived from open pollination of the maternal hermaphrodite expressed hermaphroditism (Kurahashi and Kimura, 2002). In 2003, we planted 38 and 47 clones produced from two (A and B, respectively) of the four hermaphroditic offspring with 13 and 14 clones of two normal males (C and D, respectively) in an experimental field in Ibaraki (36°0'N, 140°7'E), central Japan. The field was isolated from other natural *S. subfragilis* trees by a distance of over 500 m.

We sprayed giberic acid on the A clones on 19 and 27 July 2005 to facilitate flowering. In April 2006, we observed abundant hermaphroditic flowers on the A clones and collected the fruits produced from open pollination on 3 May 2006. Thus, the seed parent was A, and the candidate pollen parents were A in selfing and B, C and D in outcrossing. In May 2006, we sown seeds extracted from the friuts in pots with expanded vermiculites and began to incubate them at 20/25°C air temperature and 75% relative humidity with 15-h light duration. On 28 August 2006, we transplanted 204 seedlings (progeny) raised from the seeds to the experimental field at intervals of 0.5 m in seven rows separated by 1.5 m.

### Sibling identification

In June 2010, we collected leaves from 167 survivers of the 204 planted progeny, the seed parent (A) and the other three candidates of pollen parents (B, C and D). After the collection, we extracted total DNA from the leaves using the DNeasy Plant Mini Kit (Qiagen) to determine pollen parents and identify sibship among the progeny. We determined genotypes of the 167 progeny and the four candidate parents at eight nuclear microsatellite loci: Cha472, Cha522, Cha579 and Cha591 (Hoshikawa et al., 2009) and Sh034, Sh058, Sh123 and Sh124 (Kikuchi et al., 2005). We performed PCR amplification at the loci and electrophoresis of the amplified products following the methods described by Hoshikawa et al. (2009) using an ABI PRISM 3100-Avant Genetic Analyzer and the GeneScan 3.1 analysis software (Applied Biosystems). Among the eight loci, two loci (Cha579 and Sh034) were monomorphic, but the other six loci were polymorphic.

Every progeny had at least one of alleles of the seed parent (A) at every locus, confirming the mother-offspring relationship (Table 1). There were four candidate parent pairs: selfing of parent A (A × A), outcrossing with the other hermaphrodite B (A × B), outcrossing with male C (A × C) and outcrossing with male D (A × D). Unique alleles at Sh123 and Cha522 distinguished parent pairs A × B and A × C from others (Table 1). Genotypes at Sh124 discriminated parent pairs A × A and A × D (Table 1). Therefore, we identified parent pairs of 165 of the 167 progeny based on genotypes of the six polymorphic loci, although we did not find pollen parents of the other two progeny within the candidate parent pairs (Additional file 1). Based on genotypes of parents A and B and their progeny, parent B had a null allele at Sh124 (Table 1).

**Table 1:** **Genotypes at polymorphic nuclear microsatellite loci in seed parent A and candidate pollen parents B, C and D (upper rows) and genotypic frequency of progeny in sibling groups of parent pairs (lower rows)**

	Locus (Genotype and frequency)											
Parent	Cha472		Cha522		Cha591		Sh058		Sh123		Sh124	
A (hermaphrodite)	165.165		064.086		132.138		244.250		159.159		119.123	
B (hermaphrodite)	153.165		080.086		138.138		250.272		178.178		121.999	
C (male)	153.165		078.082		132.138		244.266		159.159		121.123	
D (male)	153.165		080.080		132.134		244.272		159.159		121.121	
Sibling group	Cha472	Freq.	Cha522	Freq.	Cha591	Freq.	Sh058	Freq.	Sh123	Freq.	Sh124	Freq.
Self (A x A)	165.165	110	064.064	**33**	132.132	36	244.244	23	159.159	110	119.119	19
			064.086	**65**	132.138	50	244.250	64			119.123	61
			086.086	**12**	138.138	24	250.250	23			123.123	30
Cross_H (A x B)	153.165	10	064.080	11	132.138	16	244.250	7	159.178	31	119.999	7
	165.165	21	064.086	3	138.138	15	244.272	6			119.121	7
			080.086	9			250.250	12			121.123	11
			086.086	8			250.272	6			123.999	6
Cross_M (A x D)	153.165	13	064.080	11	132.132	11	244.244	10	159.159	24	119.121	14
	165.165	11	080.086	13	132.134	5	244.250	5			121.123	10
					132.138	3	244.272	5				
					134.138	5	250.272	4				

Allele 999 at Sh124 indicates a null allele. Bold frequencies indicate significant (*P* < 0.042) segregation distortion in multiple *χ*^2^ tests.

### Sex expression

In April of each year from 2008 to 2012, we observed the survival and flowering of the 204 planted progeny as well as the sex expression of the flowering progeny. Hermaphrodites had female, male and bisexual inflorescences, two of the three types of inflorescences or only bisexual ones. Bisexual inflorescences had both female and male flowers at various positions and compositions, as observed in other willow species (Rottenberg, 2007). We divided the hermaphroditic expression of each progeny every year into three classes: female-biased, unbiased and male-biased hermaphroditism based on the number of female, male and bisexual inflorescences. According to the records of sex expression of individual 2- to 6-year-old progeny, we categorized the progeny into three sexes: female (only female expression was observed), male (only male expression was observed) and hermaphrodite (other cases were observed) (Additional file 1). We further divided the hermaphrodites into sub-categories based on the frequency of years when they flowered in the female, male and hermaphroditic classes (Additional file 1).

### Fitness components

We measured six fitness components in growth, survival and fertility.

Because the progeny usually had multiple stems due to sprouting, we measured the total length (m) and the basal girth (cm) of every stem of each progeny in September 2010. In terms of growth, we obtained the maximum stem length (m) and the total basal area (cm^2^) for the 165 4-year-old progeny whose sibships were identified (Additional file 1). We recorded survival in April of each year from 2010 to 2012, when 35 of the 165 progeny had died (Additional file 1).

To assess male fertility, we measured the pollen germination proportion. We collected flowering shoots from three to five male and two hermaphroditic progeny in each sibling group in April 2011 (Additional file 1). We sampled four male inflorescences from the shoots of each progeny and collected pollen from the inflorescences. We scattered the pollen on a 2% agarose gel plate (1 cm square) with 20% sucrose and 100mg/L boric acid, followed by incubation in a Petri dish for 24 h at 25°C air temperature and 95% relative humidity. On the gel plate for each progeny, we counted germinated and non-germinated pollen grains (200 to 600 grains in total). To assess female fertility, we estimated the number of ovules in each flower and the seed set in each fruit. We collected fruiting shoots from three to six female and two to six hermaphrodite progeny in each sibling group in June 2012 (Additional file 1). From the shoots of each progeny, we sampled eight fruits, each of which had two carpels. We counted the numbers of aborted embryos and sound seeds in both carpels under a binocular. Sound seeds were green in color and > 1 mm in length, while aborted embryos were transparent or pale in color and < 1 mm in length. We assumed that the number of ovules was the sum of the number of aborted embryos and the number of sound seeds. Thus, we defined the seed set as (the numbers of sound seeds)/(the number of ovules).

### Data analysis

We tested segregation distortion at segregated loci in each sibling group using *χ*^2^ test, chisq.test(x) in R 2.14.1 statistical language (R Development Core Team, 2011). *P*-values in the multiple tests were adjusted using p.adjust(x, method = “holm”).

We tested differences in the sex ratio (female:male:hermaphrodite) among the sibling groups with the *χ*^2^ tests using chisq.test(x).

We fit the fitness components of the progeny to four generalized linear models with effects of sibling group, sex and their interaction (1), only both effects of sibling group and sex (2) and only either effect of sibling group (3) or sex (4). We tested the statistical significance of the effects of sibling group, sex and their interaction with likelihood ratio tests using the *χ*^2^ approximation for models 4 and 2, 3 and 2 and 2 and 1, respectively. In the models, we applied a gamma distribution with a log link function to the maximun stem length and the total basal area using glm(x, family = Gamma(link = “log”)) and a biomial distribution with a logistic link function to the survival rate using glm(x, family = binomial(link = “logit”)) in R 2.14.1. We fit mixed models with the three fixed effects and a random effect of progeny to the pollen germination proportion and the seed set and applied a binomial distribution with a logistic link function to the models using glmmML(x, family = binomial(link = “logit”)). We fit the mixed models to the number of ovules and applied a poisson distribution with a log link function to the models using glmmML(x, family = poisson(link = “log”)). We performed the likelihood ratio test for outputs of two models, y1 and y2 < - glm(x), using anova(y1, y2, test = "Chisq") and for y1 and y2 < - glmmML(x) using a *P*-value = 1 - pchisq(y1$deviance - y2$deviance, y1$df.deviance - y2$df.deviance).

We evaluated inbreeding depression for fitness components from mean measurements of selfed progeny *W*_s_ and those from progeny crossed with a hermaphrodite *W*_oh_ or those from progeny crossed with a male *W*_om_ in the following form:

$$\delta_{h}=1-W_{s}/W_{\mathrm{oh}}\left(W_{s}\;\leq \;W_{\mathrm{oh}}\right),W_{\mathrm{oh}}/W_{s}\;-\;1\;\left(W_{s}>W_{\mathrm{oh}}\right);$$

$$\delta_{m}=1-W_{s}/W_{\mathrm{om}}\left(W_{s}\;\leq \;W_{\mathrm{om}}\right),W_{\mathrm{om}}/W_{s}\;-\;1\;\left(W_{s}>W_{\mathrm{om}}\right).$$

The magnitude of inbreeding depression *δ*_h_, in comparison with the progeny derived from outcrossing with a hermaphrodite, would be underestimated because the hermaphroditic parents were siblings (Kurahashi and Kimura, 2002). On the other hand, *δ*_m_, in comparison with the progeny derived from outcrossing with an unrelated male, would be an appropriate estimate of inbreeding depression. To estimate the mean, median and 95% range of *δ*_h_ and *δ*_m_, we obtained 10,000 replications of the mean of bootstrap samples from the measurements at the given sample sizes using mean(sample(x, length(x), replace = TRUE)) in R 2.14.1.

## Results

### Sex expression

Based on genotypes at the seven microsatellite loci, we divided the 165 genotyped progeny into three sibling groups: 110 progeny derived from selfing of hermaphroditic parent A (Self), 31 progeny from outcrossing with the other hermaphroditic parent B (Cross_H) and 24 progeny from outcrossing with male parent D (Cross_M). We did not find any progeny derived from outcrossing with male parent C, probably due to its less-overlapping flowering time with seed parent A.

Most (13 of 14) segregated loci in the sibling groups did not significantly deviated from the Mendelian segregation ratio (*P* > 0.626) although significant segregation distortion was found only at locus Cha522 in Self (*P* < 0.042) (Table 1).

The sex ratio differed significantly among the three sibling groups (*χ*^2^ = 22.9, *df* = 4, *P* < 0.001). The sex ratio (female:male:hermaphrodite) was characterized by the dominance of hermaphrodites (27:17:66) in Self, the scarecity of females (3:16:12) in Cross_H and the relatively even expression (9:8:7) in Cross_M (Table 2). Across the sibling groups, the sex expression of hermaphrodites in 2- to 6-year-old progeny was biased to male because more hermaphroditic progeny showed both male and hermaphroditic expression (52) than both female and hermaphroditic expression (10) (Table 2). Among 242 cases of hermaphroditic expression observed during the 5 years across the sibling groups (196 in Self, 30 in Cross_H and 16 in Cross_M), 111 cases were classified as male-biased expression (84 in Self, 19 in Cross_H and 8 in Cross_M), whereas 67 cases were classified as female-biased expression (61 in Self, 2 in Cross_H and 4 in Cross_M), and the rest were classified as unbiased expression. Thus, the hermaphroditic expression of individual progeny in each year was also biased to male.

**Table 2:** **Sex expression of sibling groups (Self: progeny derived from selfing, Cross_H: progeny from outcrossing with another hermaphrodite and Cross_M: progeny from outcrossing with a male) in** ***Salix subfragilis***

Sex			Sibling group								
	Sub-category		Self			Cross_H			Cross_M		
Female (F)			27			3			9		
Male (M)			17			16			8		
Hermaphrodite (H)			66			12			7		
	Only F and M years			1			1			0	
	Only F and H years			8			1			1	
		Biased to F years			2			1			0
		Biased to H years			6			0			1
	Only M and H years			39			8			5	
		Biased to M years			15			3			4
		Biased to H years			24			5			1
	Only H years			18			2			1	
Total			110			31			24		

In hermaphroditic expression, category and sub-category are recognized based on the frequency of sex expression in 2- to 6-year-old progeny. In hermaphrodites, for example, sub-category "only F and M years" means that there are both years when female expression was observed and years when male expression was observed. In "only F and H years", for example, "biased to F years" means that number of years when female expression was observed are more than that when hermaphroditic expression was observed.

### Inbreeding depression

**Figure 1:**
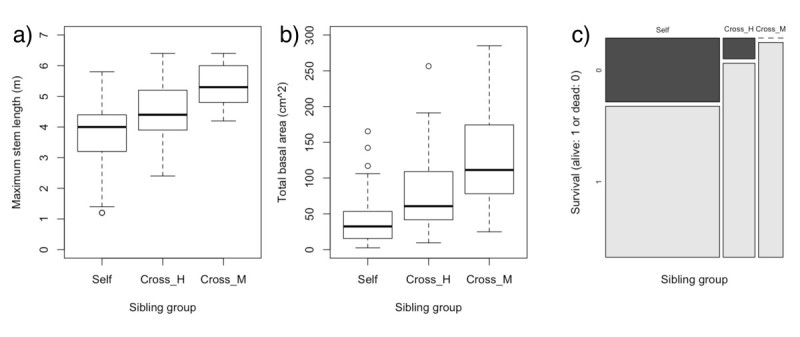
**Inbreeding depression in growth and survival in** ***Salix subfragilis*** **.** Differences in growth (maximum stem length **(a)** and total basal area **(b)** of 4-year-old progeny) and survival (survival rate **(c)** during ages from 4 to 6 years) among sibling groups (Self: progeny derived from selfing, Cross_H: progeny from outcrossing with a sibling hermaphrodite and Cross_M: progeny from outcrossing with an unrelated male).

The fitness components of growth and survival, the maximum stem length (m) and the total basal area (cm^2^) in 4-year-old progeny and the survival rate during ages from 4 to 6 years were siginificantly different among the sibling groups (*χ*^2^ > 16.8, *df* = 2, *P* < 0.001) but were not significantly dependent on sex (*χ*^2^ < 3.2, *df* = 2, *P* > 0.203) or interactions between sibling group and sex (*χ*^2^ < 5.7, *df* = 4, *P* > 0.228) (Table 3). The growth and survival components were lowest in Self, highest in Cross_M and intermediate in Cross_H (Figure 1). On the other hand, the fertility components, the pollen germination proportion, the number of ovules and the seed set, were not affected by the effects of sibling group (*χ*^2^ < 5.2, *df* = 2, *P* > 0.076), sex (*χ*^2^ < 1.6, *df* = 1, *P* > 0.194) or their interactions (*χ*^2^ < 4.1, *df* = 2, *P* > 0.132) (Table 3).

**Table 3:** **Log-likelihood ratio tests for effects of sibling group (Self: progeny derived from selfing, Cross_H: progeny from outcrossing with another hermaphrodite and Cross_M: progeny from outcrossing with a male), sex (female, male and hermaphrodite) and their interaction on fitness components of growth, survival and fertility in** ***Salix subfragilis***

Fitness component	Effect (difference in deviance, ***df***, ***P***-value)								
	Sibling group			Sex			Interaction		
Maximum stem length (m)	58.18	2	< 0.001	3.19	2	0.203	5.64	4	0.228
Total basal area (cm^2^)	68.06	2	< 0.001	1.04	2	0.594	3.08	4	0.544
Survival rate	16.85	2	< 0.001	0.19	2	0.910	5.31	4	0.257
Pollen germination proportion	5.16	2	0.076	1.69	1	0.194	4.05	2	0.132
No. of ovules	0.35	2	0.839	0.24	1	0.626	0.06	2	0.970
Seed set (no. of seeds/no. of ovules)	4.59	2	0.101	0.53	1	0.468	3.04	2	0.219

The magnitude of inbreeding depression *δ*_h_, when the fitness components were compared between sibling groups Self and Cross_H, tended to be smaller than that of *δ*_m_, when compared between sibling groups Self and Cross_M (Table 4). Among the six fitness components, the maximum stem length (m), the total basal area (cm^2^) and the survival rate had higher magnitudes of inbreeding depression (0.16 < *δ*_h_ < 0.51 and 0.29 < *δ*_m_ < 0.70) than the pollen germination proportion and the seed set (0.06 < *δ*_h_ < 0.09 and 0.11 < *δ*_m_ < 0.16) (Table 4). The number of ovules had no significant magnitude of inbreeding depression (-0.05 < *δ*_h_, *δ*_m_ < 0.05) (Table 4).

**Table 4:** **Bootstrap estimates of inbreeding depression (reduction in fitness components of selfed progeny compared with Cross_H: progeny derived from outcrossing with a sibling hermaphrodite and Cross_M: progeny from outcrossing with an unrelated male) in growth, survival and fertility in** ***Salix subfragilis***

Fitness component	***δ***_h_(compared with Cross_H)				***δ***_m_(compared with Cross_M)			
	Mean	Median	95% range		Mean	Median	95% range	
Maximum stem length (m)	0.165	0.166	0.087	0.236	0.293	0.294	0.244	0.340
Total basal area (cm^2^)	0.504	0.510	0.343	0.627	0.693	0.695	0.602	0.764
Survival rate	0.217	0.222	0.069	0.336	0.297	0.297	0.216	0.387
Pollen germination proportion	0.066	0.066	0.011	0.118	0.110	0.111	0.068	0.151
No. of ovules	0.000	0.000	-0.043	0.044	0.000	0.000	-0.044	0.043
Seed set (no. of seeds/no. of ovules)	0.088	0.090	0.009	0.162	0.156	0.156	0.098	0.211

## Discussion

In dioecious plants, the presence of rare hermaphroditic morphs including monoecious and polygamous plants, which is known as subdioecy, has been reported (Dorken and Barrett, 2004). The occurrence of rare hermaphrodites in subdioecious populations is thought to be the final transition from either gynodioecy or androdioecy toward dioecy, the initial breakdown of established dioecy or a mere developmental noise. On the other hand, stable subdioecy potentially has adaptive significance in relation to variation in abiotic and biotic environments including the availability of resources and mates (Dorken and Barrett, 2004). In willows, hermaphroditic plants and populations have been found in some species but are exceptional in this genus with strict dioecy (Smith, 1940; Rohwer and Kubitzki, 1984; Falinski, 1998; Rottenberg, 2007). Thus, exceptional hermaphrodites in willows are likely to originate from the initial breakdown of the dioecious system or developmental instability in sex expression.

An extraordinary hermaphroditic tree was found in a natural *S. subfragilis* population (Kurahashi and Kimura, 2002). Two hermaphroditic offspring of the tree, which were at least half-sibs, were used in this study. Sex expression and fitness components were measured in the progeny of one of the two hermaphrodites and compared among the progeny derived from selfing or outcrossing with the other sibling hermaphrodite or an unrelated male. The Mendelian segregation at most loci suggests that sex-related genes and deleterious alleles of progeny were normally inherited from their parents (Table 1). Sex expression and inbreeding depression are known to be influenced by maternal effects and dependent on the individuality of maternal trees (Alström-Rapaport et al., 1997; González-Varo and Traveset, 2010). However, because of the rarity of hermaphrodites in willows, we investigated a single maternal tree. With respect to the importance of maternal effects on sex ratio and inbreeding depression, the findings in this study have to be carefully applied to other populations of *S. subfragilis* and other *Salix* species.

### Sex expression

Progeny of the maternal hermaphrodite showed not only female or male but also hermaphroditic expression (Table 2). Sex expression of the hermaphroditic 2- to 6-year-old progeny was biased to male (Table 2). The hermaphroditic expression of individual progeny in each year was also male-biased in terms of floral abundance. Male-biased hermaphrodites, or inconstant males, were observed in offspring from crosses of some *Salix* species, and most hermaphrodites reverted to male expression with age (Mosseler and Zsuffa, 1989). The existence of inconstant males in many dioecious willows suggests that the male is heterozygous at sex determination loci (Charlesworth, 2002) although the heterozygous sex is unclear in *Populus*, the sister genus of *Salix* (Paolucci et al., 2010). In willows, inconstant males suggest that the supression of female expression in a male genotype is labile, depending on environmental conditions (Mosseler and Zsuffa, 1989). In the studied *S. subfragilis*, genetic, epigenetic and cytoplasmic changes in the factor supressing female expression in males may be responsible for the occurance of hermaphrodites.

Hermaphroditism recurred in progeny, suggesting that the bisexuality is genetically based (Table 2). Maternal epigenetic or cytoplasmic factors alone are not sufficient to explain the difference in the sex ratio among the sibling groups (Table 2) because the maternal tree was identical among the sibling groups. Thus, genotypes at nuclear loci seem to be responsible for sex expression in the progeny. The identity of allele combinations may also affect sex expression because the progeny derived from selfing and outcrossing of the hermaphroditic parents exhibited different sex ratios (*χ*^2^ = 18.0, *df* = 2, *P* < 0.001) (Table 2). Nearly half (66 of 110) of the selfed progeny were hermaphrodites, suggesting that the identical combination of parental alleles in progeny reproduced the hermaphroditism of the parent.

An explicit genetic model for the transition from hermaphroditism to dioecy through gynodioecy was proposed using both male- and female-sterility loci linked closely (Charlesworth and Guttman, 1999). First, a hermaphroditic population, where a male-fertile allele (*S*^M^) and a female-fertile allele (*Su*^f^) are fixed at the two linked loci, is invaded by a recessive male-sterility allele (*S*^m^), resulting in the establishment of *X* (*S*^m^*Su*^f^) haplotypes and homozyogous females (*XX*). Next, a dominant female-sterility allele (*Su*^F^) spreads among hermaphrodites, conferring *Y* (*S*^M^*Su*^F^) haplotypes and heterozyogous males (*XY*). The breakdown of this system occurs with recombination of *X* and *Y* haplotypes in a male genome, leading to revesion to the hermaphroditic haplotype *H* (*S*^M^*Su*^f^). This model was applied to *Sagittaria latifolia*, a species with monoecious and dioecious populations, and successfully explained the sex ratio observed in various crosses (Dorken and Barrett, 2004). In that model, crosses between hermaphrodites (*HH* or *XH*) result in either 100% hermaphrodites or 25% females and 75% hermaphrodites. This prediction disagrees with the results of this study in terms of not only hermaphrodites and females but also males found in the progeny derived from selfing and outcrossing of the hermaphrodite parents. However, with regard to the inconstant male expression, males in the progeny can be regarded as hermaphrodites. Under this assumption, segregation between females and males plus hermaphrodites (27:(17 + 66)) was close to 1:3 (*χ*^2^ = 0.012, *df* = 1, *P* = 0.912) in the selfed progeny (Table 2). In *Urtica diota*, which consists of females, males and monoecious hermaphrodites, selfing of hermaphrodites resulted in a ratio of one female to three male/hermaphroditic plants (Glawe and Jong, 2008). Thus, our findings suggest a genetic basis of the extraordinary hermaphroditism found in *S. subfragilis*, which are consistent with some models for the genetic factors in subdioecious sex expression.

### Inbreeding depression

Selfing in the maternal hermaphrodite were frequent among progeny derived from open pollination. This result suggests the absence of barriers to self-fertilization, such as dichogamy and self-incompatibility. These barriers do not function and are likely to be lost in dioecious willows. Unfortunately, we did not measure fitness components at the early stages of the life cyle, such as embryo development, seed germination and seedling performance, because we did not conduct controlled pollination. Inbreeding depression at the early life-cycle stages differed between outcrossing and selfing plants (Goodwillie and Knight, 2006; Ishida, 2008). Selfing plants tend to have lower magnitudes of inbreeding depression at the early stages than at the late stages of the life cyle because deleterious alleles expressed at the early stages are effectively purged through inbreeding, while those at the late stages are difficult to purge even in inbred populations (Husband and Schemske, 1996). On the other hand, high magnitudes of inbreeding depression were expected at the both early and late life-cycle stages in predominantly outcrossing species because opportunities to purge deleterious alleles are rare (Husband and Schemske, 1996). Therefore, although abundant selfed progeny were found, the magnitude of inbreeding depression could have been as high at the early stages as at the late stages, when fitness components were measured as growth, survival and fertility of trees.

As we expected, *δ*_h_ was lower than *δ*_m_ in most fitness components (Table 4), suggesting that the relatedness of parents affects the fitness of their progeny (Charlesworth and Willis, 2009). The magnitude of inbreeding depression was higher in growth and survival than in fertility (Table 4). According to a meta-analysis of inbreeding depression in various fitness components, the magnitude of inbreeding depression was highest in growth (plant biomass), lowest in fertility (pollen and ovule traits) and intermediate in survival (Angeloni et al., 2011), which is consistent with our results. Inbreeding depression in progeny of *Populus nigra* derived from full-sib crosses, which is expected to be lower than that in selfed progeny of *S. subfragilis*, was measured in the height and diameter of stems of 2- and 3-year-old progeny (Benetka et al., 2008). The magnitudes of inbreeding depression were from 0.046 to 0.112 in the stem height and from 0.209 to 0.395 in the stem cross-sectional area in *P. nigra*, nearly half *δ*_m_ in *S. subfragilis* (Table 4). Thus, the inbreeding depression observed in *S. subfragilis* has a reasonable magnitude based on the previous studies.

We did not find any evidence that differences in sex affected the fitness components (Table 4), although sexes differed in fitness-trait performance in *S. glauca* (Dudley, 2006). In our study, the seed set did not significantly differ between females and hermaphrodites, the former of which was outcrossing exclusively, but the latter had the potential of selfing (Table 4). This result, together with the abundant selfed progeny found in our samples, suggests that selfing hardly affects seed production in *S. subfragilis*. In contrast to our results, a substantial reduction in the pollen viability and the fruit set was observed in extraordinary hermaphrodites of *P. euphratica* and *S. acmophylla* (Rottenberg, 2000; 2007). In a population of *S. acmophylla*, all individuals were polygamous and likely to reproduce vegetatively (Rottenberg, 2007). Thus, variation in reproductive systems among populations seems to affect the fertility of different sexes.

## Conclusions

The findings of this study suggest a genetic basis of extraordinary hermaphroditism and a substantial magnitude of inbreeding depression in survival and growth in normally dieocious *S. subfragilis*. The inbreeding depression leads to selection against inbred progeny of hermaphrodites and exclusion of mutant alleles responsible for the hermaphroditism. These processes are likely to maintain strict dioecy in willows.

## Electronic supplementary material

Additional file 1:**Genotypes, sex expression and fitness components of 167 progeny.**(CSV 14 KB)

Below are the links to the authors’ original submitted files for images.

Authors’ original file for figure 1
