# Supplementary figures and images for: Sex expression and inbreeding depression in progeny derived from an extraordinary hermaphrodite of Salix subfragilis
Source: Bot Stud. 2014 Jan 14;55:3. doi: 10.1186/1999-3110-55-3 (PMC5432739; doi:10.1186/1999-3110-55-3)

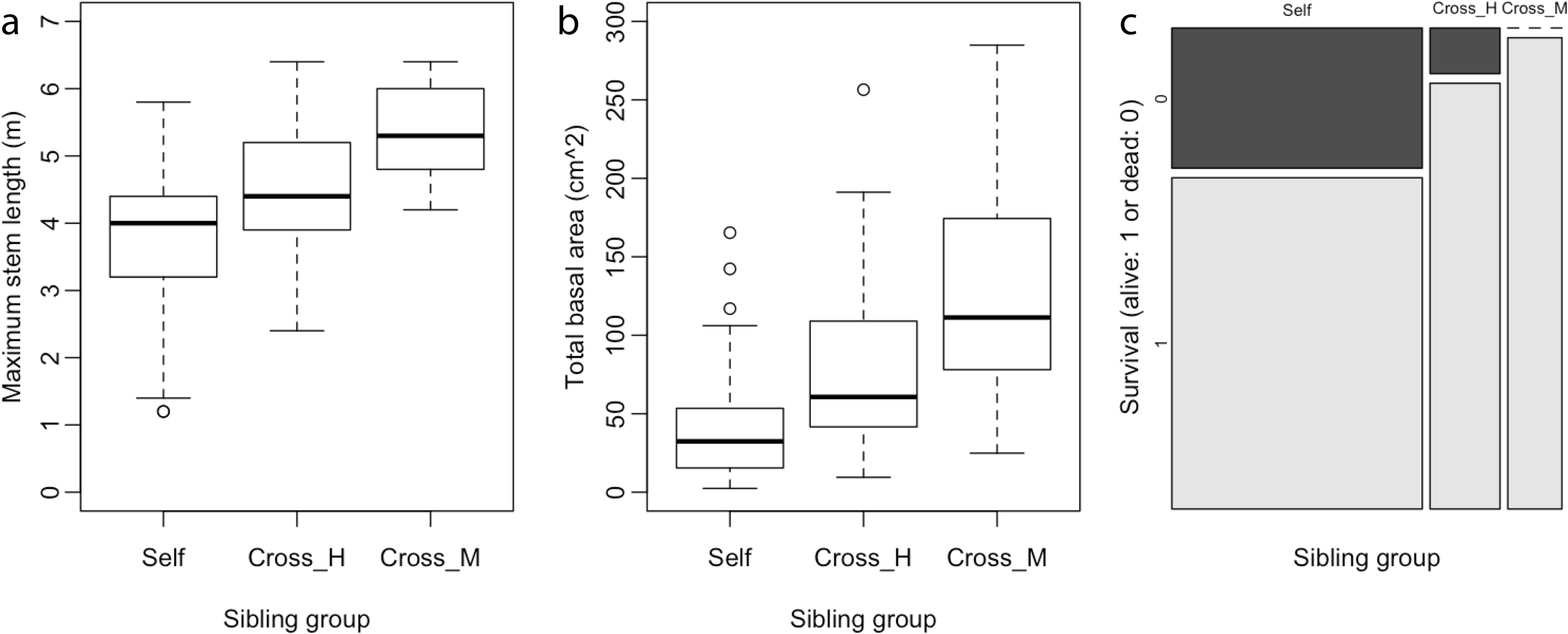

Supplement: Supplementary file 2 — Authors’ original file for figure 1 [file 40529_2013_54_MOESM2_ESM.tif]
